# A multi-species evaluation of digital wildlife monitoring using the Sigfox IoT network

**DOI:** 10.1186/s40317-023-00326-1

**Published:** 2023-03-25

**Authors:** Timm A. Wild, Louis van Schalkwyk, Pauli Viljoen, Georg Heine, Nina Richter, Bernd Vorneweg, Jens C. Koblitz, Dina K. N. Dechmann, Will Rogers, Jesko Partecke, Nils Linek, Tamara Volkmer, Troels Gregersen, Rasmus W. Havmøller, Kevin Morelle, Andreas Daim, Miriam Wiesner, Kerri Wolter, Wolfgang Fiedler, Roland Kays, Vanessa O. Ezenwa, Mirko Meboldt, Martin Wikelski

**Affiliations:** 1 Department of Migration, Max Planck Institute of Animal Behavior, 78315 Radolfzell, Germany; 2 Department of Biology, University of Konstanz, 78464 Constance, Germany; 3 Product Development Group Zurich (pd|z), ETH Zürich, Leonhardstr. 21, 8092 Zurich, Switzerland; 4 Centre for the Advanced Study of Collective Behaviour, University of Konstanz, 78464 Constance, Germany; 5 Section for Zoology, Natural History Museum of Denmark, University of Copenhagen, 2100 Copenhagen, OE Denmark; 6 Department of Ecology and Evolutionary Biology, Yale University, 165 Prospect Street, New Haven, CT 06511 USA; 7 Department of Game Management and Wildlife Biology, Czech University of Life Science, 165 00 Prague, Czech Republic; 8 Institute of Wildlife Biology and Game, Department of Integrative Biology and Biodiversity Research, University of Natural Resources and Life Sciences (BOKU), 1180 Vienna, Austria; 9 Vulpro NpC, Vulture Programme, Plot 121 Boekenhoutkloof Road, Rietfontein, 0216 South Africa; 10 Zoo Salzburg, 5081 Anif, Austria; 11 Department of Agriculture, Land Reform and Rural Development, P.O. Box 12, Skukuza, 1350 South Africa; 12 Department of Veterinary Tropical Diseases, Faculty of Veterinary Science, University of Pretoria, Private Bag X04, Soutpan Road, Pretoria, 0110 South Africa; 13 Scientific Services, South African National Parks, Skukuza, 1350 South Africa; 14 North Carolina Museum of Natural Sciences, Raleigh, NC 27601 USA; 15 Department of Forestry and Environmental Resources, North Carolina State University, Raleigh, NC 27607 USA

**Keywords:** Animal tracking, Movement ecology, Telemetry, Biologging, LPWAN, LoRa, Wireless sensors, Embedded systems, Onboard processing, Sigfox

## Abstract

Bio-telemetry from small tags attached to animals is one of the principal methods for studying the ecology and behaviour of wildlife. The field has constantly evolved over the last 80 years as technological improvement enabled a diversity of sensors to be integrated into the tags (e.g., GPS, accelerometers, etc.). However, retrieving data from tags on free-ranging animals remains a challenge since satellite and GSM networks are relatively expensive and or power hungry. Recently a new class of low-power communication networks have been developed and deployed worldwide to connect the internet of things (IoT). Here, we evaluated one of these, the Sigfox IoT network, for the potential as a real-time multi-sensor data retrieval and tag commanding system for studying fauna across a diversity of species and ecosystems. We tracked 312 individuals across 30 species (from 25 g bats to 3 t elephants) with seven different device concepts, resulting in more than 177,742 successful transmissions. We found a maximum line of sight communication distance of 280 km (on a flying cape vulture [*Gyps coprotheres*]), which sets a new documented record for animal-borne digital data transmission using terrestrial infrastructure. The average transmission success rate amounted to 68.3% (SD 22.1) on flying species and 54.1% (SD 27.4) on terrestrial species. In addition to GPS data, we also collected and transmitted data products from accelerometers, barometers, and thermometers. Further, we assessed the performance of Sigfox Atlas Native, a low-power method for positional estimates based on radio signal strengths and found a median accuracy of 12.89 km (MAD 5.17) on animals. We found that robust real-time communication (median message delay of 1.49 s), the extremely small size of the tags (starting at 1.28 g without GPS), and the low power demands (as low as 5.8 µAh per transmitted byte) unlock new possibilities for ecological data collection and global animal observation.

## Background

Over the past decades, humans have interconnected vehicles, shipping containers, city infrastructure, and other objects of interest by wireless technology, forming digital networks that provide insights into the ‘lives’ of our man-made tools. Within the internet of things (IoT) [1, 2] sensor data of tagged objects are autonomously collected and distributed for commercial or industrial purposes such as tracking delivery items or remotely monitoring temperatures of cooling systems. Ecologists and conservationists ask similar questions related to position, movement, surrounding environment, welfare, and dynamics, putting free-roaming animals in the limelight instead of commodities [3]. Because of the similarity of data needed to answer such questions, emerging technologies from the IoT are trickling into animal-borne biotelemetry (e.g., LoRa [4–7], Bluetooth 5 [8], WiFi [9]) and transforming wildlife research [10–12]. However, solutions for tracking ocean containers or garbage cans do not necessarily transfer to uncontrollable and unpredictable wild animals that spend lifetimes in the harsh and rapidly changing conditions of natural environments, leading to different requirements for the technologies used.

Sigfox is a Low Power Wide Area Network (LPWAN) that has attracted the attention of IoT companies and scientists [13, 14]. This technology consists of a global network of terrestrial base stations (soon, potentially on satellites [15]). Tracking devices integrate small, commercially available Sigfox radio chips that wirelessly transmit units of several bytes of sensor data to the network (uplink) and or receive short commands (downlink). The communication on specific frequency bands (e.g., 868 MHz in Europe) is optimised for low energy consumption while achieving kilometre-wide transmission ranges [14]. Sigfox is not the only LPWAN network, and there are comparable systems like LoRa, NB-IoT and LTE-M [16]. Despite LoRa being recently proven to be a valuable tool in wildlife research [4–7], other LPWANs, including Sigfox, remain largely untested on wild animals. While commercial Sigfox deployments on livestock show promise [17], biologging on wildlife may bring more challenging demands on accessibility, robustness, network coverage, antenna performance, power consumption, cost and mass. Sigfox was recently tested on urban gulls, but transmission performance was not assessed [18], making it difficult to understand the feasibility of broader use in wildlife. Because of the unique challenges posed by biologging in natural conditions, we argue that devices and deployments on wildlife should be studied in a category of their own, the ‘internet of animals’ [3, 10].

Slight differences in technologies and capabilities of LPWANs can massively affect the applicability of particular systems and infrastructure for the internet of animals and biosphere monitoring. As an example, Sigfox offers increased transmission ranges that allowed to cover Belgium (30,600 km^2^) with only seven base stations [19, 20]. Such transmission capabilities become crucial for tracking projects in large remote wilderness areas. Here, we evaluate Sigfox as a solution for real-time wildlife tracking across continents, habitats, and a broad range of focal taxa. We have developed a multi-tag and multi-attachment toolbox, consisting of several electronic devices that exponentially expand the diversity of trackable animals when combined with modular housings and mounting tools. Our method includes onboard processing of sensor data to overcome data size limitations, tailoring tag deployments to specific ecological questions, and detecting events in real-time (e.g., mortality, geo-fences, or dangerous conditions). Further, we test the proprietary Sigfox Atlas Native system, allowing low-power positional estimates of tagged animals without GPS, enabling smaller tags.

In this large-scale study, we deployed 312 Sigfox devices on 30 species in 12 countries, collecting 177,742 total biologically relevant multi-sensor data messages. Our toolbox ranges from tiny 1.28 g collars that reveal previously unknown migration paths of common noctule bats (*Nyctalus noctula*) through Europe and continues with songbird backpacks (2.55 g) that send high-frequency environmental and activity data to the Sigfox network. We also demonstrate how the same technology can be embedded in solar-powered collars, suitable for use on mid-sized species. Our ear tag design (32.4 g) allowed us to tag and successfully track a range of larger mammals, including white rhinoceroses (*Ceratotherium simum*) and African buffaloes (*Syncerus caffer*). We also developed a 56.5 g prototype that can harvest kinetic energy and is suitable for long-term deployments on photophobic species (e.g., wild boar [*Sus scrofa*]), where solar-powered devices are not an option. All collected data are automatically archived on Movebank [21–23] and are accessible a few seconds after the message transmission via the Animal Tracker app in the field by researchers, conservation managers or citizen scientists [10, 21]. Furthermore, commands and configurations can be sent to the Sigfox tracking tags via downlink messages, thus enabling a two-way data stream. Near real-time tracking information enables not only the study of an entire new set of free-roaming animals, but also a new set of management responses, as demonstrated in same-hour responses by veterinarians to snaring events, reducing human-caused mortality among critically endangered African wild dogs (*Lycaon pictus*) in Kruger National Park [24, 25].

## Materials and methods

### About the Sigfox network

**Fig. 1:**
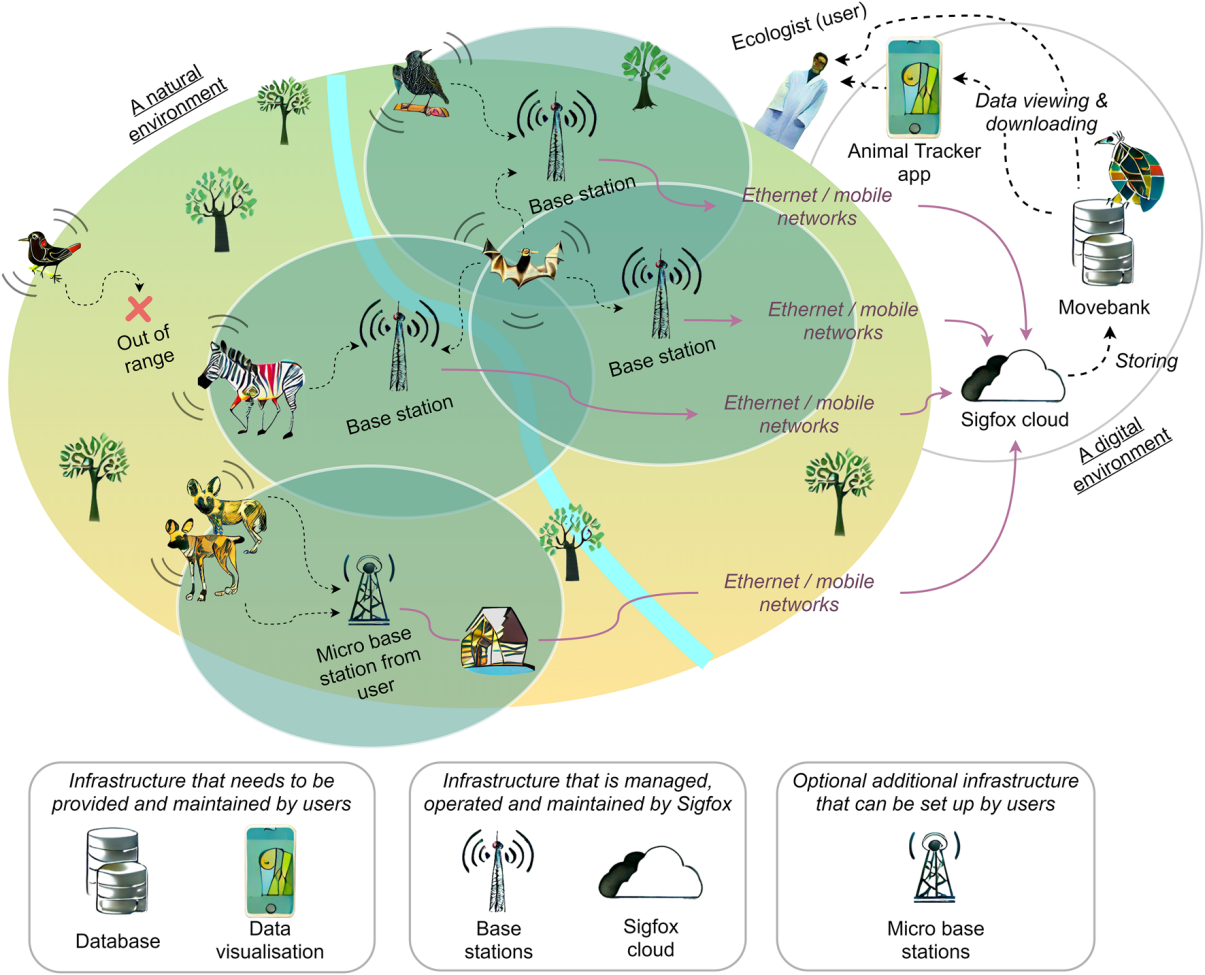
Infrastructure of a Sigfox network to track free-roaming animals. Animal-borne tags transmit messages to nearby base stations. Each base station requires permanent internet access and sends incoming messages to the Sigfox cloud for temporary storage. Then, data are forwarded to Movebank for persistent storage. Researchers can access data via the Animal Tracker app or via the Movebank website (www.movebank.org [26])

The Sigfox infrastructure consists of many terrestrial base stations that are connected to a central database via the internet (Fig. 1). We implemented an interface to Movebank (www.movebank.org [26]), a database for persistent data archiving used widely by ecologists and conservationists. Collected data are automatically linked to Movebank studies that are managed by the associated researchers, and include meta-data (e.g., deployment times or animal descriptions) and access management. Researchers can either use the visualisation and export tools of Movebank or the Animal Tracker smartphone app for immediate data access (e.g., to locate an animal when working in the field), or link from Movebank’s programming interface into any other database system such as EarthRanger (www.earthranger.com [27]). The growing global base station network is managed, operated, and maintained by Sigfox (coverage maps available at www.sigfox.com/coverage [28]), but users can extend the network themselves (e.g., in remote natural areas) by setting up small, commercially available base stations with limited range (micro base stations). It is also possible to order the deployment of full-sized base stations through Sigfox. Each base station needs permanent internet access to participate (e.g., via cellular networks or satellite-based connectivity). Due to regional differences in legislation the global network is currently split into seven radio configurations (RC1–RC7). Each configuration covers one or more countries. The configurations vary for example in transmission centre frequencies (between 865 and 923 MHz) or maximum transmission power (16 or 24 dBm). These differences have an impact on the device design (e.g., requiring differently tuned antennas or RC-specific radio chips) and potentially on region-specific tag performance. Sigfox transmissions are digitally modulated by Binary Phase Shift Keying (BPSK), have a bandwidth of 100 Hz and a maximum data rate of 100 bps [20].

### Remote data collection with Sigfox

Wildlife trackers need to integrate a licensed radio chip and an antenna with region-specific tuning to participate in the Sigfox network. Devices are then registered at the Sigfox backend with a unique id hardcoded into the radio chips. Currently available Sigfox-compatible radio chips can be as small as 5 × 5 × 0.6 mm and as light as 33 mg (STMicroelectronics STM32WL series). In small electronic devices both the antenna type (e.g., chip, whip, patch, flex, or helix) and the device design (e.g., size of the ground plane or housing material) strongly influence wireless transmission performance (e.g., maximum communication range). Sigfox allows devices to send up to six uplink messages within a one-hour period (i.e. 140 messages per day). Each uplink message contains meta-data (e.g., the receiving time and a consecutive message number) and up to 12 bytes of application-specific data (the ‘payload’). For instance, an uplink message could contain a single GPS fix consisting of 4-byte latitude, 4-byte longitude, and a 4-byte timestamp with up to 7 decimal digits of GPS precision and second-level time precision. Uplink messages are not acknowledged by the network, so on-animal devices cannot determine whether a message was successfully received by Sigfox infrastructure. To know exactly how many transmission events (and resulting data) are lost in a given Sigfox deployment, we calculated the transmission success rate on animal-borne Sigfox devices by comparing the consecutive number of the last received message with the total number of received messages. Devices can receive up to 8 bytes of downlink data from the network, up to four times per day. Devices acknowledge the receipt of a downlink message. We performed power consumption measurements of both uplink and downlink transmissions with an ON Semiconductor AX-SIP-SFEU-1-01-TX30 Sigfox chip, an ON Semiconductor NCP170AMX330TBG 3.3 V low dropout voltage regulator, and an Otii Arc source measurement unit set at 3.75 V.

### GPS-less geo-locating of animals with Atlas Native

Sigfox offers a proprietary geo-location service, Atlas Native, which estimates the device position (latitude, longitude, accuracy range in m) for each received message. Sigfox claims an accuracy in the range of 1 to 10 km in 80% of the messages [29]. These positional estimates are calculated in the Sigfox cloud by a proprietary closed-source algorithm that uses the received signal strengths of messages and known positions of receiving base stations. The concept is similar to VHF-based trilateration [30], but allows additional sensor data to be transmitted in the message payload (max. 12 bytes). Atlas Native does not require any additional energy to that of sending a message. The integration of satellite navigation (e.g., GPS) requires additional electronic components and increases mass and power consumption of tracking devices. We experimentally evaluated the actual accuracy of Atlas Native on animals by enabling the service on devices that also integrate GPS and compared both positional estimates with each other.

### Portfolio of Sigfox animal tracking tags

Our proposed range of animal-borne tags consists of four custom-designed electronic circuit boards that differ in size and sensor composition. Each design allows different question- and species-tailored energy use and sensor deployments, but all implement Sigfox for remote data retrieval. For Sigfox connectivity, the tags either integrate a SEONG JI SFM10R1, a SEONG JI SFM10R4, an LPRS eRIC-SIGFOX-RCZ1 or an ON Semiconductor AX-SIP-SFEU-1-01-TX30 Sigfox chip, combined with either a quarter wave monopole whip antenna (86.3 mm in length for 868 MHz), a helix antenna, or a flexible antenna with IPEX MHF connector.

**Fig. 2:**
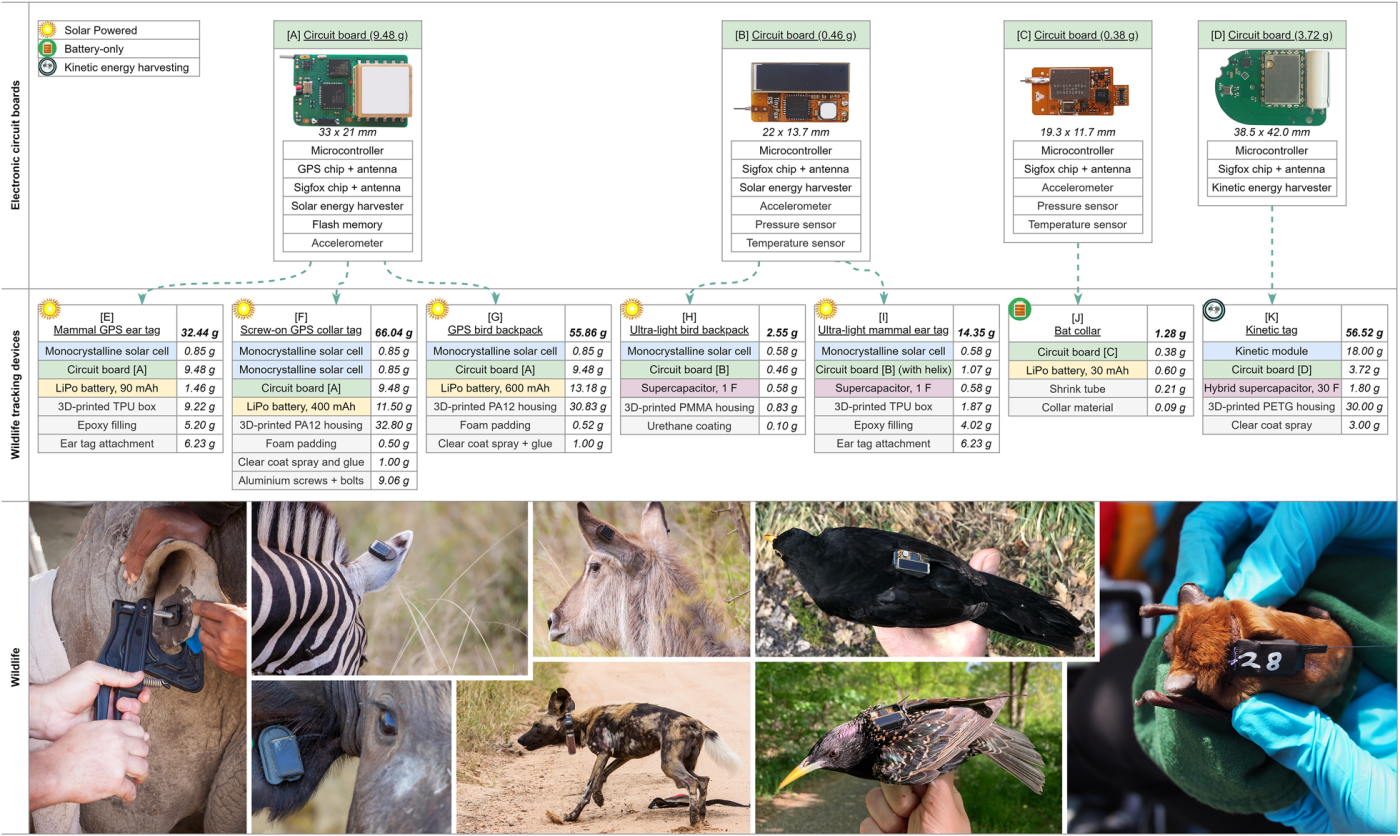
Overview of Sigfox tag prototypes for wildlife tracking. The tag designs (**E**–**K**) are based on four different electronic circuit boards (**A**–**D**). We integrated the circuit boards into differently designed tracking devices, consisting of a power source (LiPo battery: **E**, **F**, **G**, **J**, supercapacitor: **H**, **I**, **K**), an optional source for generating power (solar cell: **E**–**J**, kinetic harvesting module: **K**), a 3D-printed housing (thermoplastic polyurethane [TPU]: **E**, **I**, polymethyl methacrylate [PMMA]: **H**, nylon polymer [PA12]: **F**, **G**, polyethylene terephthalate glycol [PETG]: **K**), an attachment method (ear tag: **E**, **I**, collar: **F**, **J**, backpack: **G**, **H**, as counterweight: **K**), and waterproofing (epoxy: **E**, **I**, clear coat spray: **F**, **G**, **K**, shrink tube: **J**, urethane coating: **H**)

*Circuit board design [A]* (Fig. 2a) (9.48 g) is programmed to transmit positional estimates of an onboard GPS unit (Quectel L80-M39) via the Sigfox network. Circuit board [A] also integrates an accelerometer (Bosch BMA400) for basic activity measures, and a harvesting circuit (Analog Devices ADP5091) that recuperates energy from a monocrystalline solar cell into a lithium-polymer (LiPo) battery. The number of Sigfox messages per day depends on how much sunlight the devices are exposed to. We used this circuit board design to evaluate Atlas Native's accuracy by comparing GPS positions to Atlas Native positions when recorded at the same time.

*Circuit board [B]* (Fig. 2b) (0.46 g) is optimised for lower mass and delivers positional estimates through Atlas Native instead of GPS. Circuit board [B] records and transmits environmental data (temperature, barometric pressure [Measurement Specialties MS5637], sunlight exposure) and movement-related metrics, based on onboard-processed accelerometer data from a MEMSIC MC3635. Here we used a surface-mounted monocrystalline solar cell (ANYSOLAR Ltd KXOB25-12X1F) to charge a 1 F supercapacitor (Kyocera AVX SCCQ12E105PRB), which serves as the sole power source. The number of Sigfox messages per day depends on how much sunlight the devices are exposed to.

*Circuit board [C]* (Fig. 2c) (0.38 g) is even lighter and integrates the same sensors as circuit board [B], but is powered by a small LiPo battery instead. After activation, the tag transmits Sigfox messages in a configurable interval until the battery becomes empty. For field deployments on bats, we configured the devices to transmit four Sigfox messages per day.

*Circuit board [D]* (Fig. 2d) (3.72 g) comprises a novel harvesting circuit that recuperates kinetic energy into a lithium supercapacitor. In the current design of circuit board [D] no onboard sensors are integrated, and positional estimates are derived from the Sigfox Atlas Native location service. In the next version, we plan to integrate a GPS module and an accelerometer for added utility. The number of Sigfox messages per day depends on how much devices are moved.

Using the four circuit boards (Fig. 2a–d) as a basis for our investigation of Sigfox for ecological inquiries, we created seven deployable tag designs (Fig. 2e–k). Each design consisted of one of the circuit boards (Fig. 2a–d), a power source, an optional method to recover energy, and waterproof housing. The final tags were then attached as ear tags (14.35–32.44 g), collars (1.28–66.04 g) or backpacks (2.55–55.86 g).

### Onboard processing of sensor data

Given the limited data transmission capacity of Sigfox, we implemented processing algorithms to turn raw sensor data into purposeful metrics. This method of irreversible compression (also referred to as edge computing) has proven valuable on animals [31, 32]. Depending on the tag type and configuration, one or more of these metrics are then transmitted as part of the payload of a Sigfox message. From a 3.4-s long 3-axis 54-Hz acceleration burst, we calculated the vector of the dynamic body acceleration (VeDBA, according to [33]), the number and average amplitude of zero crossings on the *Z*-axis (according to [34]), percentual activity within the last 24 h (by comparing VeDBA values of the burst recordings of the last 24 h against a programmable threshold), pitch, and roll. Pitch and roll were only estimated when the dynamic acceleration was low (VeDBA(t) < 51 mg). We summarised the data from an onboard temperature sensor (Measurement Specialties MS5637), which was recorded every 60 s, and determined the minimum and maximum temperatures of the last 24 h.

## Results

### Energy considerations of devices on animals

**Fig. 3:**
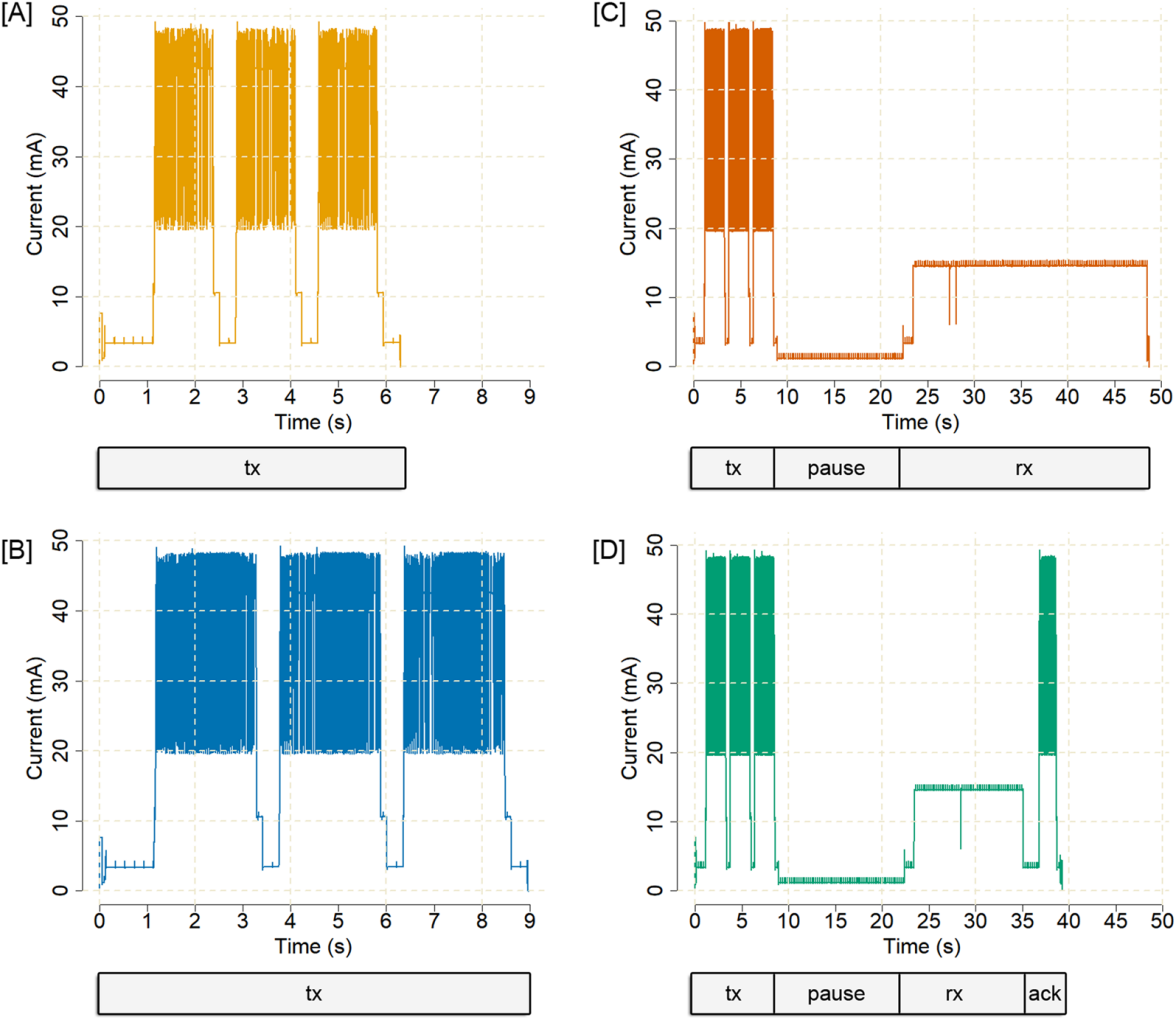
Evaluation of the power consumption of four different types of Sigfox messages. We measured the power consumption and required time of a 1-byte uplink RC1 Sigfox message (**A**), a 12-byte uplink RC1 Sigfox message (**B**), a 12-byte uplink RC1 Sigfox message followed by a failed downlink transmission (**C**) and a 12-byte uplink RC1 Sigfox message followed by a successful downlink transmission (**D**) with an ON Semiconductor AX-SIP-SFEU-1-01-TX30 Sigfox chip, an ON Semiconductor NCP170AMX330TBG 3.3 V low dropout voltage regulator, and an Otii Arc source measurement unit set at 3.75 V. The Sigfox transmission protocol includes three consecutive repetitions of the same message (tx) to increase the probability of transmission success. Some chips allow for decreasing the number of repetitions, which decreases power consumption, but affects transmission robustness. Based on Sigfox infrastructure density and tag-specific transmission success, it may be advantageous to increase or decrease repetitions; dynamic evaluation of whether towers are near or far could help maximise battery lifespan and data acquisition. The length of the reception window (rx) varies based on the response time of the base station(s), which has a non-deterministic effect on power consumption. Successfully received downlinks are acknowledged by the devices (ack)

The power consumption of a 1-byte RC1 Sigfox uplink message was, on average, 24.7 mA for 6.3 s (i.e. 43.2 µAh per byte) (Fig. 3a). A 12-byte RC1 Sigfox uplink message required an average current of 28.1 mA for 8.97 s (i.e. 5.8 µAh per byte) (Fig. 3b). Maximum current peaks were 49.3 mA (measured at a sample rate of 4000 samples per s). As a result, longer Sigfox messages were more energy efficient as they used less battery capacity per transmitted byte. The power consumption of a bidirectional transaction (a 12-byte RC1 Sigfox uplink message followed by an 8-byte downlink transmission attempt) was 13.3 mA for 39.28 s on average when a base station responded (Fig. 3d) and 13.1 mA for 48.7 s when no base station could be reached (Fig. 3c). It should be noted that the power consumption of chips of other RC zones (RC2–RC7) might differ from our measurements due to different communication frequencies and different transmission powers. With Sigfox transmissions, there is no additional power consumption between messages. Sending N messages requires exactly N times as much energy as a single message, regardless of the interval at which messages are sent.

We experimentally confirmed on six devices (circuit board [C]) that a micro-sized 30 mAh LiPo battery (0.6 g) can send an average of 240 12-byte messages, which included additional energy needed for operating a microcontroller, a temperature sensor, and an accelerometer. Assuming a transmission success rate of 50%, this corresponds to 1440 bytes of remotely available data for researchers per device (e.g., 240 6-byte GPS fixes or 720 2-byte VeDBA measurements [excluding additional power needed for the sensors]). For example, a 480 mAh LiPo battery (8 g) would allow the transmission of an average of more than 6800 12-byte messages (at a transmission success rate of 50%: 40,800 bytes of ecological data [e.g., 6800 6-byte GPS fixes] per device). When animals were regularly exposed to sunlight, the energy source of a tag could be charged with a solar cell and an onboard energy harvesting circuit (circuit boards [A] and [B]). The generated current of a custom-built monocrystalline solar cell (SolarC, 31 × 20 × 0.7 mm, 0.85 g) in combination with an e-peas AEM10941 energy harvester was 7.34 mA in direct sunlight and 1.01 mA in the shadow of a tree on a sunny day. Neglecting other electrical loads, the energy harvested in direct sunlight was equivalent to one 12-byte Sigfox message every 34.1 s. As an alternative to solar power, we used a kinetic harvester to transform animal movement into electrical energy (circuit board [D]). When attached to the underside of a dog harness, the average generated energy on a pet dog (*Canis familiaris*) was enough to transmit 11 2-byte Sigfox messages per day (including additional energy needed for operating a microcontroller).

### Field performance on animals around the world

**Fig. 4:**
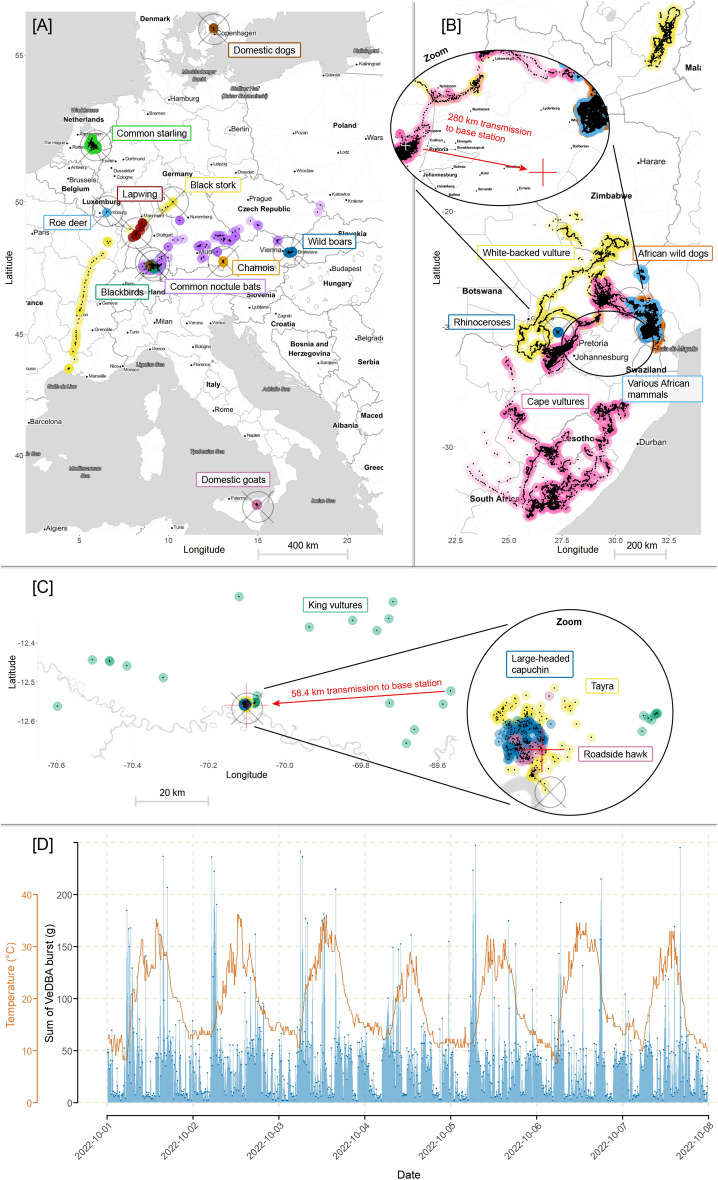
Remote data collection with Sigfox in Europe (**A**), Southern Africa (**B**) and the Amazon basin in Peru (**C**). Coloured dots show locations from which on-animal data was received via the Sigfox network. Crossed circles indicate field sites. The red crosses in **B**, **C** mark locations of selected Sigfox base stations for the exemplary visualisation of transmission distances. Plot **D** shows onboard calculated VeDBA and temperature measurements on an Impala, recorded at 10-min intervals and transmitted via Sigfox in addition to the positional estimates (GPS, Atlas Native)

With our device portfolio, we performed case studies on 312 individuals of 30 species in 12 countries, resulting in 177,742 successfully transmitted Sigfox messages under real field conditions (Table 1) (Fig. 4). This corresponded to more than 2,000,000 bytes of remotely retrieved data. We did not evaluate residual signal strength indicators (RSSI) or signal-to-noise ratios (SNR), as they are not directly comparable to other transmission technologies and aim at the same and most important question of whether a message was successfully received or not (i.e. the transmission success rate). The overall average transmission success rate equalled to 56.2% (SD 27.2).

**Table 1:** Evaluation of the performance of Sigfox-enabled tracking devices (RC1 and RC4) on various species

Class	Species (a)	Tag type(s) (b)	No. of deployments	Avg. deployment duration (days) (c)	Avg. transmission success rate (%) (d)	Avg. received messages per day per capita	Avg. total received messages per capita	Total received messages	Max. transmission range observed (km) (e)	Avg. receiving base stations (f)	Median accuracy atlas native (km) (g)
Flying, migration, Europe	Black stork (*Ciconia nigra*)	[E] (h)	1	10	87.6	13.40	134	134	N/A	N/A	N/A
	Common starling (*Sturnus vulgaris*)	[H]	1	42	42.4	30.40	1277	1277	N/A	3.33	N/A
	Common noctule bat (*Nyctalus noctula*)	[J]	18	14	62.6 (SD 22.4)	2.89	34	618	N/A	3.61	N/A
Flying, migration, Southern Africa	Cape vulture (*Gyps coprotheres*)	[G]	17	41	73.7 (SD 14.7)	12.23	609	10,369	280	5.42	21.6
	White-backed vulture (*Gyps africanus*)	[G]	1	117	61.5	26.51	3102	3102	186	3.73	44.4
Flying, non-migratory, European low mountain ranges and Alpine foreland	Blackbird (*Turdus merula*)	[H]	2	105	96.7 (SD 3.3)	3.30	381	761	N/A	5.29	N/A
	Lapwing (*Vanellus vanellus*)	[H]	2	32	60.7 (SD 37.3)	1.89	32	63	N/A	4.38	N/A
Flying, non-migratory, Amazon rainforest	King vulture (*Sarcoramphus papa*)	[G]	2	27	47.9 (SD 22.1)	1.88	59	118	58	1.00	37.2
	Roadside hawk (*Rupornis magnirostris*)	[G]	1	61	93.7	3.15	192	192	1	1.00	0.4
Non-flying, enclosure, city	Chamois (*Rupicapra rupicapra*) (1)	[E]	2	101	98 (SD 0.7)	13.03	1317	2633	132	4.38	2.4
	Chamois (*Rupicapra rupicapra*) (2)	[I]	2	191	99.9 (SD 0.1)	2.77	527	1054	N/A	4.39	N/A
	Domestic Dog (*Canis familiaris*)	[K]	3 (i)	5	52.5 (SD 20.5)	3.66	18	53	N/A	1.09	N/A
Non-flying, enclosure, shrublands and volcanic land	Domestic goat (*Capra hircus*)	[E], [F]	18	213	58.3 (SD 10.4)	12.58	2667	48,005	17	1.30	10.2
Non-flying, enclosure, African savannah	African buffalo (*Syncerus caffer*) (1)	[E]	30	33	97.2 (SD 3.2)	4.67	161	4850	N/A	1.13	N/A
Non-flying, free-roaming, European forest	Roe deer (*Capreolus capreolus*)	[E]	1	335	27.7	2.15	719	719	N/A	N/A	N/A
	Wild boar (*Sus scrofa*)	[E] (j)	12	33	19.8 (SD 13)	1.35	31	370	17	1.06	4.1
Non-flying, free-roaming, Amazon rainforest	Tayra (*Eira barbara*)	[E] (k)	1	39	88.7	11.26	439	439	1	1.00	0.7
	Large-headed capuchin (*Sapajus macrocephalus*)	[E] (k)	1	36	49.5	6.97	251	251	1	1.00	0.5
Non-flying, free-roaming, African savannah	African wild dog (*Lycaon pictus*)	[E], [F]	75	104	41.6 (SD 23)	4.69	400	29,991	133	1.56	14.4
	White rhinoceros (*Ceratotherium simum*) (1)	[E]	10	110	57.1 (SD 11.9)	7.23	628	6276	177	1.41	6.0
	White rhinoceros (*Ceratotherium simum*) (2)	[E]	6	7	55.2 (SD 11.7)	15.74	111	667		N/A	N/A
	White rhinoceros (*Ceratotherium simum*) (3)	[E]	51	74	54.3 (SD 24.3)	4.49	287	14,645		2.52	13.1
	African buffalo (*Syncerus caffer*) (2)	[E]	3	148	22.8 (SD 4)	0.98	122	365	115	1.54	25.9
	Cheetah (*Acinonyx jubatus*)	[E]	2	468	33.5 (SD 1.7)	2.41	1124	2248	81	1.10	17.5
	Lion (*Panthera leo*) (1)	[E]	3	24	37.1 (SD 5.5)	5.72	97	292	91	1.02	4.2
	Lion (*Panthera leo*) (2)	[E]	6	29	44 (SD 20.8)	4.84	103	617		1.33	19.3
	Black rhinoceros (*Diceros bicornis*) (1)	[E]	1	1	21.5	26.00	26	26	119	1.00	13.2
	Black rhinoceros (*Diceros bicornis*) (2)	[E]	3	107	36.4 (SD 17.4)	2.28	160	480		1.21	40.1
	Black rhinoceros (*Diceros bicornis*) (3)	[E]	5	17	53.7 (SD 26.7)	5.89	54	272		N/A	N/A
	African bush elephant (*Loxodonta africana*)	[E]	1	3	79.5	10.33	31	31	61	N/A	N/A
	Spotted hyena (*Crocuta crocuta*)	[E]	4	39	59.8 (SD 7.1)	4.11	151	605	108	1.44	14.6
	Greater kudu (*Tragelaphus strepsiceros*)	[E]	6	108	49.4 (SD 17)	4.97	563	3375	150	1.58	22.5
	Plains Zebra (*Equus quagga*)	[E]	10	133	54.1 (SD 21.9)	19.84	2919	29,185	195	1.87	17.0
	Waterbuck (*Kobus ellipsiprymnus*)	[E]	2	54	98.9 (SD 0.6)	16.85	1037	2074	83	2.96	4.1
	Impala (*Aepyceros melampus*) (1)	[E]	1	77	96.5	18.25	1405	1405	76	2.58	6.1
	Impala (*Aepyceros melampus*) (2)	[I]	1	114	69.5	32.75	3734	3734	N/A	1.77	N/A
	Blue wildebeest (*Connochaetes taurinus*)	[E]	3	171	53.7 (SD 10.8)	5.85	971	2914	118	1.37	17.0
	Giraffe (*Giraffa camelopardalis*)	[E]	4	252	35.1 (SD 17.8)	7.56	883	3532	N/A	N/A	N/A

(a) Species are listed multiple times if tracked in multiple projects, i.e. different environments; (b) tag types are described in Fig. 2; (c) some devices were still deployed and operational at the time of writing this publication; (d) calculation of the average transmission success rate and SD is based on consecutive message numbers; (e) distance calculations use the Haversine formula for great-circle distances and are only possible when devices integrate GPS and base station positions are known (otherwise N/A); (f) calculations only consider successful transmissions and are only possible when Sigfox meta-data are available (otherwise N/A); (g) accuracy calculations use the Haversine formula for great-circle distances between the GPS-derived positional estimates and the Atlas Native positional estimates (only possible on GPS-enabled devices and when Atlas Native is enabled, otherwise N/A); (h) ear tag was modified and attached to an ELSA snap-fit ring; (i) one of the two dogs was tagged twice; (j) since wild boar mainly stay in the shade, the ear tags were modified to run on battery power only (no solar cell, a 480 mAh battery instead of a 90 mAh battery); (k) ear tags were attached to collars in a modified form

**Fig. 5:**
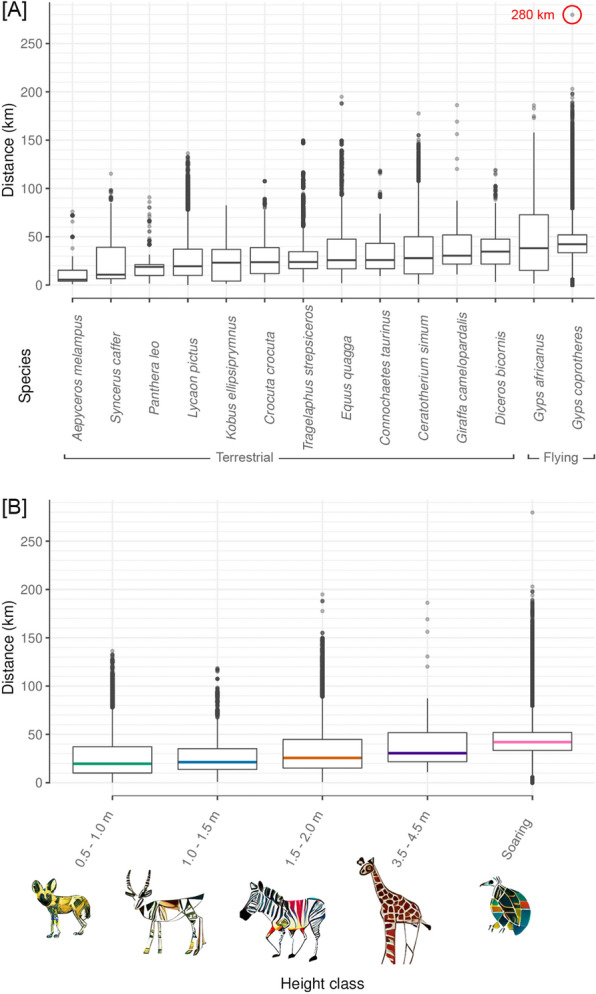
Observed transmission distances between animal-borne Sigfox tags and receiving Sigfox base stations in southern Africa. Animals are grouped by species (**A**) and average tag height above ground (**B**). Distances were determined by using the Haversine formula for the great-circle distance between the GPS-generated tag location and the known locations of southern African base stations. Low median values below 50 km do not indicate low maximum transmission distances, but rather that animals often stayed in the vicinity of certain base stations. Height class 0.5–1.0 m includes African wild dogs. Height class 1.0–1.5 m includes African buffaloes, lions, spotted hyenas, impalas and blue wildebeests. Height class 1.5–2.0 m includes black and white rhinoceroses, plains zebras, waterbucks and greater kudus. Height class 3.5–4.0 m includes giraffes. The soaring class includes cape vultures and white-backed vultures

As with all terrestrial wireless technologies, a transmission success primarily depends on the antenna type, the density of receiving stations, the topography of the area, the vegetation, the climate [35], and the habitat use of an animal. Transmission success rates on flying species were on average 14.2% higher than on non-flying species, which is due to the better radio signal propagation in open space. For example, on a flying lapwing (*Vanellus vanellus*) in Germany a single data message was received by as many as 26 base stations. We evaluated transmission ranges by using the Haversine formula for the great-circle distance between GPS locations of devices and locations of base stations (Fig. 5). We found maximum communication distances of up to 280 km on flying species (cape vultures) and up to 195 km on non-flying species (plains zebras [*Equus quagga*]). Our measurements demonstrate that a single Sigfox base station can cover a maximum of 246,300 km^2^ under optimal circumstances. We did not test the performance of Sigfox on non-flying animals in mountainous areas. We also did not evaluate the impact of climate (temperature, humidity, barometric pressure) on the transmission success rate.

For animals that stayed within a local area that was covered by at least one base station, the average transmission success rate was very high (e.g., 98.0% [SD 0.7] on chamois [*Rupicapra rupicapra*] that live in a city park in Salzburg, Austria, or 97.2% [SD 3.2] on African buffaloes that were held in enclosures in Kruger National Park), showing why this technology is increasingly being considered in livestock farming [17]. Urban areas are more densely covered with Sigfox base stations than rural ones, leading to higher transmission success rates (e.g., 96.7% [SD 3.3] on blackbirds living near the city of Konstanz, Germany). We measured an average transmission success rate of 62.8% (SD 22.4) when tracking long-distance migrations of flying species in Europe, revealing several coverage gaps of the network, especially in Eastern Europe. The transmission success rate on wild boar was comparably low (19.8% [SD 13.0]). We assume a significant impact of the vegetation type on the transmission success rate, especially as wild boar spend most of the time deep inside their dense forest habitat. In the Amazon rainforest, where a single base station was covering a local area, transmission success was variable across the three species that moved locally near the tower (hawk: 93.7%, tayra: 88.7%, monkey: 49.5%), showing that species behaviour and microhabitat use can greatly affect tag performance.

The performance of the kinetic tag (Fig. 2k) on domestic dogs was tested with a first prototypical implementation in which the antenna design was not yet optimised. We expect a performance improvement in the next development iterations. However, we can already conclude that Sigfox is a suitable technology for small devices operating on kinetic energy due to its low power consumption.

We calculated the accuracy of the Atlas Native service by comparing 87,550 Atlas Native positional estimates on 21 species with the GPS-derived positional estimates of the devices (using the Haversine formula for great-circle distances) and found an overall median accuracy of 12.89 km (MAD 5.17). Sigfox provides an estimated accuracy in m for each Atlas Native position. 48.39% of the positional estimates were within this estimated accuracy. The positional accuracy was ≤ 10 km in 31.96% of the messages, which is worse than the accuracy stated by Sigfox (≤ 10 km in 80% of the messages [29]). We were able to measure positional accuracies of up to ≤ 100 m for animals that were less than 1 km away from a base station, making Atlas Native suitable for studying habitat use when setting up site-specific base stations. Contrary to classic trilateration algorithms, Atlas Native's median accuracy did not improve with a higher number of receiving base stations (Table 2).

**Table 2:** Accuracy evaluation of Atlas Native on animals based on the number of receiving base stations

No. of receiving base stations	No. of evaluated messages	Best accuracy (km)	Median accuracy (km)
1	49,409	0.021	12.05 (MAD 3.82)
2	17,288	0.155	11.27 (MAD 4.34)
3	6006	0.141	15.19 (MAD 9.12)
4	3614	0.143	14.44 (MAD 10.61)
> 4	11,233	0.086	18.56 (MAD 12.40)
1 − ∞	87,550	0.021	12.89 (MAD 5.17)

To test the suitability of Sigfox for real-time tracking (e.g., in situ warnings of danger or mortality), we measured the time between the end of a transmission and persistent data storage of a total of 1,812,354 messages over an entire year on both deployed and undeployed tags (based on meta-data provided by the Sigfox network). The median delay was 1.49 s (not including the actual transmission time of up to 8.97 s). Responses by local managers, limited additionally by recognition and response times, were less than 30 min in some cases (e.g., helping a snared African wild dog in Kruger National Park).

### Sigfox across borders

Long-distance migrating species traverse countries (e.g., common noctule bats) and even continents (e.g., black storks [*Ciconia nigra*]). From a technological point of view these crossings pose a particular challenge for terrestrial tracking systems, as infrastructure, network operators, and legal considerations can differ significantly between countries (e.g., leading to increased international roaming costs for GSM tracking devices [36]). We found that within the same RC zone, devices were able to move across multiple country borders without an increase in power consumption, subscription cost, or message delay. This enabled for example the continuous tracking of migrating birds and bats in Europe, and chamois living on the country border between Germany and Austria. Near country borders, we observed that single messages were sometimes received by multiple base stations from multiple countries. In our tag designs we exclusively integrated RC-specific radio chips. We did not evaluate multi-RC Sigfox chips (e.g., the SEONG JI SRM100A) that allow devices to automatically switch between RCs, either based on reading base station broadcasts (a feature called Sigfox Monarch) or based on GPS-enabled geo-fencing.

### Estimating project cost

Wildlife research often operates on limited budgets. We compiled some examples of costs that should be considered when using Sigfox on animals (Table 3).

**Table 3:** Example costs of using Sigfox to track animals

Class	Cost item	Cost (USD)
Electronic Sigfox chips for tags (examples)	ON Semiconductor AX-SIP-SFEU-1-01-TX30	12
	SEONG JI SFM10R1 (RC1)/SFM10R4 (RC4)	4
	LPRS eRIC-SIGFOX-RCZ1	17
Tags (examples)	Wimbitek WIMBI SF Bird Tracker (a)	N/A
	Globalsat ST-20 (ear tag for cattle)	163
	Digitanimal Livestock GPS (collar)	180
	Sensolus TRACK 1000 (not designed to be attached to animals)	112
	Figure 2e–k (material costs only)	< 100
Base stations	Sigfox Access Station Micro SMBS-T4 (reduced range)	480
	Full-sized Sigfox Base Station (deployment costs)	> 4000 [20]
Subscription costs per tag per year (Germany) (b)	Max. 2 messages per day, Atlas Native disabled	7
	Max. 70 messages per day, Atlas Native disabled	8
	Max. 140 messages per day, Atlas Native disabled	11
	Max. 140 messages per day, Atlas Native enabled	12

(a) To our knowledge there is currently only one commercial manufacturer of Sigfox devices specifically designed to be attached to wildlife [37], but we were unable to obtain any cost information; (b) subscription costs decrease when registering more than 1000 devices

## Discussion

To address the modern challenges of global change, we need to monitor the planet at larger scales and distributed sensors are critical for this effort [3]. Here, we report on using the new generation of low-power IoT technology to network these sensors small enough to attach to animals. Our adaption of the Sigfox IoT network to animal tracking encompasses seven novel tag developments that we used to observe 312 individuals of a broad range of 30 distinct species, including both migratory and non-migratory movements. The tags varied in sensor composition, attachment method and mass (from 1.28 to 66.04 g), allowing to track small flying species with a body mass from 25 g (e.g., common noctule bats) up to some of the largest living land mammals in the world (e.g., rhinoceroses and elephants).

With power consumption as low as 5.8 µAh per transmitted byte, maximum transmission distances of up to 280 km and an overall average transmission success rate of 56.2% (SD 27.2) across species, continents, and habitats, we conclude that Sigfox has the potential to play an important role in understanding animal movement in a detailed spatial and temporal scale in near-real time. The maximum communication distance we measured on a cape vulture sets a new documented record for animal-borne data transmission with terrestrial infrastructure, enabling theoretical coverage of up to 246,300 km^2^ with a single base station. Variations in transmission performance arose from the antenna performance on tags, the density of receiving stations, the topography of a given area, vegetation, climate [35], and habitat use of an animal. Due to a very low median message delay (1.49 s), we found that the network is particularly suitable for projects where receiving real-time data is important. Speculatively, applications such as geo-fencing around protected areas, poaching surveillance, the detection of snaring and trapping events, human conflict risk and local empowerment, or automated turbine and vehicle alerts for nearby animals all could be positively affected by implementation of such a technology. The payload of up to 12 bytes in size can contain any type of raw or onboard-processed multi-sensor data (including, but not limited to, GPS, accelerometers, magnetometers, temperature sensors, and pressure sensors), which enables the collection of combined data sets that were previously impossible to get hold of. One method to circumvent the limitation of the amount of transmitted data, is question-specific onboard processing of fine scale data (e.g., from accelerometers). The Sigfox Atlas Native geo-location service is another positive aspect of this network as it allows location without GPS, enabling smaller tracking tags that can be used on a wider variety of species. Atlas Native offers lower spatial accuracies than satellite navigation (median accuracy of 12.89 km [MAD 5.17]) but allowed the development of particularly small devices (e.g., 1.28 g bat collars) due to low power demands (e.g., on average 240 positions on a 30 mAh LiPo battery [0.6 g]) and fewer electronic components. We were able to measure positional accuracies of up to ≤ 100 m for animals that were less than 1 km away from a base station, making the technology suitable to for example detect animal presence in small areas. Surprisingly, a higher base station density did not result in more accurate positional estimates. The Atlas Native error radius provided by the network was exceeded in 51.61% of the estimates. The global large-scale network coverage is managed by Sigfox and allows researchers to focus on their ecological questions instead of building and maintaining infrastructure. However, to close local gaps in coverage, the network of base stations can be extended by researchers, as long as permanent internet access can be maintained.

Terrestrial communication systems comparable to Sigfox include GSM, LoRa, LTE-M and NB-IoT. GSM is still widely being used as a transmission system for animal tracking data and a similar global study of over 1000 deployed devices found an average transmission success rate of 93.6% [38]. But GSM transmissions require comparatively large chips [39, 40], introduce high international roaming costs [36], and need about 29 times more energy compared to sending a Sigfox message [39]. LoRa enables even lower power consumption than Sigfox [4], but the transmission performance has only been evaluated theoretically, on livestock, or off-animal [4–7, 41–44]. In stationary experiments with LoRa devices, maximum transmission ranges of up to 30 km were measured [45], but we assume a significant loss of range when devices are attached to wild animals in their natural environments, as for example vegetation types and moving bodies have notable effects on the radio signal propagation [46, 47]. Other comparable LPWANs, including NB-IoT [19, 48, 49] and LTE-M [50, 51], remain unexplored on animals. Satellite-based transmission systems represent an alternative or addition to terrestrial LPWANs and are increasingly being optimised for low power consumption [52]. Before deciding on a tracking technology, we encourage scientists to carefully evaluate regional network coverage of the various systems in advance of a study, and ideally to test them on site. Furthermore, we encourage the future development of devices that can transmit across multiple networks depending on what is in range.

With technological innovations trickling into movement ecology, there is now more opportunity to study a broad range of species across a vast scale. We think that Sigfox-enabled devices can satisfy the requirements of many remote wildlife tracking studies and have the potential to partly digitalise and unify the field of animal biotelemetry. The combination of different tag designs for various species and a flexible communication network like Sigfox allows monitoring of entire ecosystems, which was previously not possible with such a level of detail at such a range. Adding short-range high-throughput communication (e.g., Bluetooth 5 [8] or WiFi [9]) to LPWANs has been explored on animals already [41] but would benefit from the increased transmission ranges of Sigfox. Our data present a current snapshot of the performance of the system. Due to the growing spatial coverage of stations, we predict that the value of this network will grow in the next few years, especially for observing long-distance migrations.

## Conclusions

In this study, we introduce an advancement to the field of animal-borne biologging by developing a custom-designed set of wildlife tracking devices with different attachment methods that use Sigfox for low-power long-range remote digital data transmission and location estimates. We analysed data from 312 tags on 30 species in 12 countries and found average transmission success rates between 19.8 and 99.9% depending on species, habitat, and network density. We measured communication distances of up to 195 km on non-flying and 280 km on flying species. Based on the results, we think that the Sigfox IoT network provides a field-ready solution for tracking a broad range of focal taxa and will help to digitalise the field of biotelemetry.

## Data Availability

The Amazon rainforest datasets are publicly available at Movebank (www.movebank.org [26]) (Movebank study ID: 2122748764). The other datasets generated and or analysed during the current study are not publicly available due to ongoing studies and to protect animals from poaching but are almost entirely archived on Movebank (Movebank study IDs: 2155070222, 1409712816, 894254831, 1365616235, 1493312931, 1296030530, 1725249380, 1431850095, 1323242594, 1732512659, 1286005281, 1291290503, 1600771155, 1670322706, 1623175929, 1323163019, 1323668146, 2057805903, 2198940839), and can be made available by the authors upon reasonable request.

## Abbreviations

SD: Standard deviation
MAD: Median absolute deviation
GPS: Global Positioning System
IoT: Internet of Things
LPWAN: Low power wide area network
LoRa: Long range
BPSK: Binary phase shift keying
NB-IoT: Narrow Band Internet of Things
LTE-M: Long-term evolution for machines
GSM: Global System for Mobile Communications
LiPo: Lithium-polymer
PMMA: Polymethyl methacrylate
TPU: Thermoplastic polyurethane
PA12: Nylon polymer 12
PETG: Polyethylene terephthalate glycol
VeDBA: Vector of Dynamic Body Acceleration
RC: Radio configuration
VHF: Very high frequency
RSSI: Residual signal strength indicators
SNR: Signal-to-noise ratio
